# Biological responses to extreme weather events are detectable but difficult to formally attribute to anthropogenic climate change

**DOI:** 10.1038/s41598-020-70901-6

**Published:** 2020-08-21

**Authors:** R. M. B. Harris, F. Loeffler, A. Rumm, C. Fischer, P. Horchler, M. Scholz, F. Foeckler, K. Henle

**Affiliations:** 1 Department of Conservation Biology, Helmholtz Centre for Environmental Research-UFZ, Permoserstr. 15, 04318 Leipzig, Germany; 2 Antarctic Climate and Ecosystems Cooperative Research Centre, University of Tasmania, Hobart, Australia; 3 Discipline of Geography & Spatial Sciences, University of Tasmania, Hobart, Australia; 4 ÖKON Ltd. Ass. for Landscape Ecology, Limnology, and Environmental Planning, Hohenfelser Str. 4, Rohrbach, 93183 Kallmünz, Germany; 5 Institute of Geosciences, Friedrich Schiller University, Burgweg 11, 07749 Jena, Germany; 6 Department Vegetation Studies, Landscape Management, German Federal Institute of Hydrology (BfG), Am Mainzer Tor 1, 56068 Koblenz, Germany; 7 German Centre for Integrative Biodiversity Research (iDiv) Halle-Jena-Leipzig, Deutscher Platz 5e, 04103 Leipzig, Germany

**Keywords:** Attribution, Climate-change ecology

## Abstract

As the frequency and intensity of extreme events such as droughts, heatwaves and floods have increased over recent decades, more extreme biological responses are being reported, and there is widespread interest in attributing such responses to anthropogenic climate change. However, the formal detection and attribution of biological responses to climate change is associated with many challenges. We illustrate these challenges with data from the Elbe River floodplain, Germany. Using community turnover and stability indices, we show that responses in plant, carabid and mollusc communities are detectable following extreme events. Community composition and species dominance changed following the extreme flood and summer heatwave of 2002/2003 (all taxa); the 2006 flood and heatwave (molluscs); and after the recurring floods and heatwave of 2010 and the 2013 flood (plants). Nevertheless, our ability to attribute these responses to anthropogenic climate change is limited by high natural variability in climate and biological data; lack of long-term data and replication, and the effects of multiple events. Without better understanding of the mechanisms behind change and the interactions, feedbacks and potentially lagged responses, multiple-driver attribution is unlikely. We discuss whether formal detection and/or attribution is necessary and suggest ways in which understanding of biological responses to extreme events could progress.

## Introduction

Extreme climatological events are important drivers associated with ongoing anthropogenic climate change^1,2^. As mean climate conditions change, the frequency and intensity of extreme events such as droughts, heatwaves and floods are also projected to increase^3^. Extreme events can result in changes to the distribution of populations of individual species or community-level responses such as changes to species richness, composition and/or dominance e.g.^4–6^. These changes may be long lasting or irreversible if competitive interactions are altered, especially when species become (locally) extinct, or with recurring extreme events e.g.^7–10^. Extreme biological responses to individual extreme weather events are already being observed in many ecosystems around the world^9,11–17^, and interest in attributing such responses to anthropogenic climate change is increasing^1^.

However, it has been questioned whether it is possible (or indeed necessary) to formally detect and attribute biological responses to anthropogenic climate change (henceforth “climate change”) as is done in the climate system^18, 19^. The IPCC defines “detection” as a demonstration that the likelihood of occurrence of an observed change is significantly different from that due to natural internal variability, without attempting to explain the causes of the observed change^20,21^. In contrast, “attribution” attempts to identify the most likely causes for the detected change with some defined level of confidence. Attribution requires that the detected change is consistent with the responses of the system to the given drivers, and not consistent with alternative plausible explanations^22,23^.

**Figure 1:**
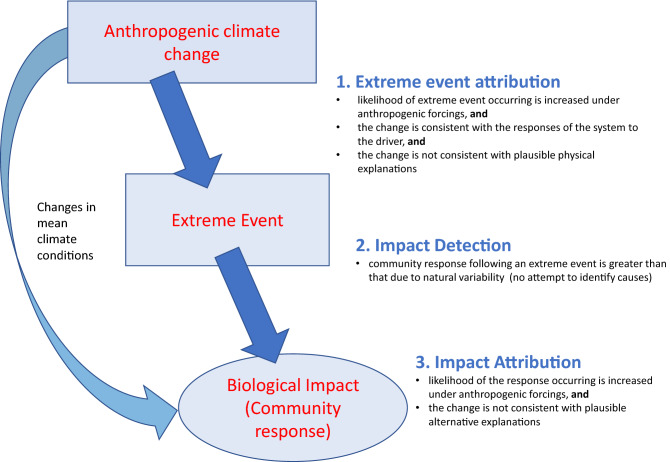
The three steps required to formally attribute biological impacts to extreme events under climate change.

Studies documenting biological responses to climate change are therefore generally not formal detection or attribution studies^24^, with rare exceptions, e.g.^25^. They describe biological changes, and either make no attempt to attribute the cause, or speculate that the cause of the observed response is climate change^26^. To attribute a biological response to an extreme event caused by climate change requires a three-step, joint attribution^27^. First, the likelihood of the extreme event would have to be attributed to climate change, then the biological response detected, and finally, attributed to the extreme event (Fig. 1). Thus, the causative starting point is the anthropogenic drivers of change and the end-point is the biological response to an extreme weather event^19^.

Floodplains provide an ideal opportunity for the study of biological impacts of extreme events. Regular floods are a natural and essential feature of the ecosystem, driving erosion and sedimentation, carbon sequestration, nutrient retention and cycling^28,29^, and leading to highly dynamic seasonal and inter-annual fluctuations in ground and surface water availability^30^. Floodplain plant and animal communities are therefore adapted to periodic flooding events. The biota tends to be characterised by features enabling populations to survive or recolonise after regular flooding events, but also to endure long dry periods, such as an annual life cycle, ability to over-winter in a dormant stage, small body size or high dispersal capability^31–35^.

However, under accelerating climate change, the nature of extreme events in floodplains appears to be changing, with altered regimes of intensity, timing and duration of floods and greater incidence of other extreme conditions such as heatwaves and drought. In recent years, several floods have occurred in Germany that have been both extreme in terms of the height and duration of flooding and unusual in terms of timing (Supplementary Figure S1). Over the same period, extreme heat events and drought periods have occurred. In August 2002, an extreme summer flood occurred on the River Elbe following high rainfall during July and August. In July and August 2003, extreme high temperatures led to the hottest summer on record in Europe since at least 1,540^36^. Mean summer temperatures exceeded the 1961–90 mean by 3 °C^36^. Additionally, there was very low precipitation from May to August 2003. The year 2005 was notable because there was no spring flood. In 2006, a spring flood was followed by a summer heatwave. In 2010, several floods recurred throughout the year, with the water level peaking above the historical mean water level at least 12 times (Supplementary Figure S1). In 2013, an early summer flood followed an intense rainfall event in which some areas received the usual monthly precipitation amount within 1 or 2 days^37^. In this year, and in 2010, summer heatwaves also occurred.

**Figure 2:**
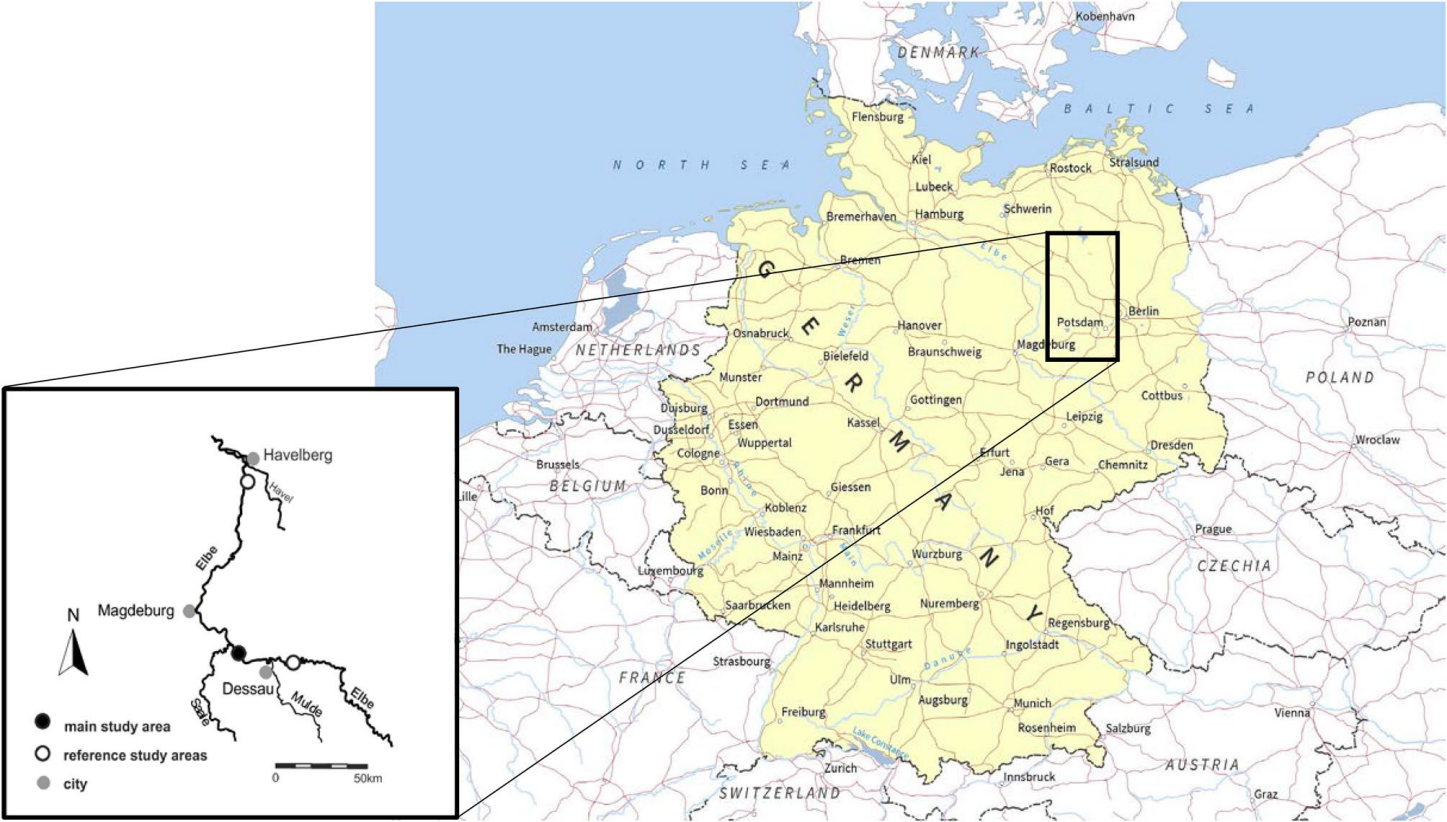
Location of the three sampling sites in Central Germany ( Source: MapsWire).

Our objective is to illustrate the challenges associated with detection and attribution of community responses to climate change, with the focus on extreme events. Shifts in the underlying climate suitability may have occurred with recent changes in mean climate, but the magnitude of transient changes during extreme events can exceed long-term changes projected for the end of century under a high emissions scenario. We test both components of change using historical climate data and biological data spanning 17 years from floodplain grasslands on the Elbe River, Germany (Fig. 2). We apply a climatological definition of extreme events and include both meteorological events, such as high temperatures and precipitation, and the resulting flooding events. First, we describe changes to climate in the study region from 1961 to 2015, and the extreme events that have occurred since 2000. We assess the literature to identify whether any of these events can be attributed to recent changes in climate. Then we describe changes to the vegetation, carabid beetle and mollusc communities over time (1998–2014). We use three taxonomic groups to highlight the challenges involved with detecting responses in different assemblages. We focus on community turnover and stability indices to quantify temporal variation in the relative abundance of species in the community and identify if any community response can be attributed to the extreme events. Finally, we discuss whether formal detection and/or attribution are needed and provide suggestions as to how understanding of biological responses to extreme events could be advanced.

## Results

### Air temperature

**Figure 3:**
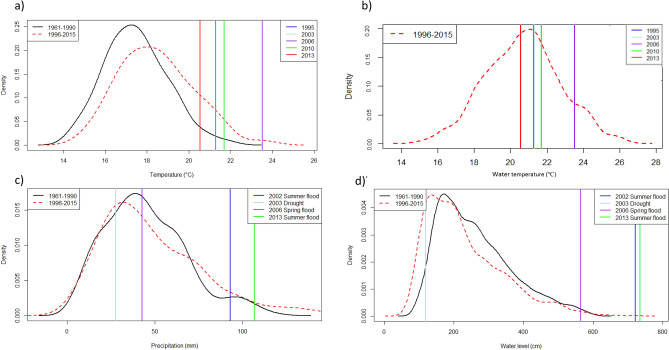
Probability distribution functions of climate variables for the historical period (1961–1990) and the sampling period (1996–2015): (**a**) mean monthly summer air temperature. Maximum summer (June, July, August) values for recent heat events are displayed as vertical lines. Note that the 2003 and 2010 lines overlap; (**b**) observed daily summer water temperatures. Data not available for the historical period; (**c**) mean monthly precipitation. Mean values for the two months preceding each extreme rainfall event are displayed as vertical lines, as follows: 2002 summer flood (July, August); 2003 drought (July, August); 2006 spring flood (February, March); 2013 summer flood (May, June); (**d**) annual water level. Maximum values for the two months preceding each extreme event are displayed as vertical lines, as follows: 2002 summer flood (July, August); 2003 drought (July, August); 2006 spring flood (February, March); 2013 summer flood (May, June). Means for each variable are based on the weather stations outlined in Supplementary Information S1.

There has been warming over the region since the historical period, with mean monthly temperatures showing an increase of approximately 1.5 °C after seasonal variability is accounted for using time series decomposition (See Supplementary Material, Supplementary Figure S2). Summer temperatures during the recent period were, on average, 1.5 °C warmer than during the earlier period (1961–1990 = 17.46 ± 1.5 °C, 1996–2015 = 18.54 ± 1.8 °C) (Fig. 3a). An increase in variance has accompanied the shift in the mean, resulting in substantially more summer days at the extreme hot end of the distribution (mean maximum summer values 1961–1990 = 21.82 °C, 1996–2015 = 23.52 °C).

Heatwaves occurred in the years 1995, 2003, 2006, 2010, 2013, with high temperatures recorded in June, July and August (only July and August in 2003). With the exception of 2013, the maximum daily summer temperature during all recent heat events exceeded the 99th percentile (21.0 °C) of the historical distribution and thus represent extreme climatological events. Although the highest observed temperatures were recorded in 2006 and 2010, there were more extremely hot days in 2003 (43 days in summer), with three heatwave events during which five or more consecutive days exceeded the 99th percentile of the historical period (1961–1990) (21.0 °C) (Table 1).

**Table 1:** Total number of extreme heat days and number of heatwave events (defined as days above the 99th percentile of the historical period (1961–1990) and periods of five or more consecutive extreme heat days, respectively).

Year	Total number of days > 21.0 °C	Number of heatwave events (# of days in each)
2003	43	3 (5,12,15)
2006	34	3 (6,5,15)
2010	28	2 (8,10)
2013	22	2 (12,6)

### Water temperature

Without historical time series of water temperature it is not possible to assess the long-term trends or if water temperatures during recent events are extreme in relation to the historical period. However, the maximum Summer water temperature during the 2003 drought (26.0 °C), the 2002 (24.3 °C) and 2013 (22.2 °C) summer floods exceeded, or equalled, the 99th percentile of the sampling period (22.2 °C) (Fig. 3b).

### Precipitation

There is no significant difference in mean monthly precipitation between the historical and recent years (1961–1990 = 44.7 ± 23.3 mm; 1996–2015 = 48.28 ± 28.9 mm), however a slight positive trend in precipitation since 1998 is apparent once the seasonal variability is removed (Supplementary Material, Supplementary Figure S3). Additionally, there has been an increase in the occurrence of intense rainfall events, and the seasonal cycle is weaker in the current period (Fig. 3c). The increase in extreme rainfall is most apparent in summer (seasonal probability distribution functions shown in Supplementary information, Supplementary Figure S5).

Mean monthly precipitation prior to the 2002 flood (92.9 mm) exceeded the 90th percentile (74.2 mm) and values preceding the 2013 flood (106.7 mm) approached the 99th percentile (107.0 mm). The 2006 flood was a more typical spring flood, with a mean value (42.7 mm) just above the 50th percentile (42.4 mm).

### Elbe water level

The mean annual water level of the Elbe River in the study region has not changed significantly between the historical and current periods (1961–1990 = 260.76 ± 105.1 cm; 1996–2015 = 231.03 ± 111.0 cm), although a slight decline in the mean monthly water level since the 1980s is apparent once the seasonal variability is removed (Supplementary Material, Supplementary Figure S4). The extreme high values of the distribution have been extended in the recent period (Fig. 3d). Maximum water levels during the floods of 2002 (721.0 cm), 2006 (565.0 cm) and 2013 (734.5 cm) all exceeded the 99th percentile of the historical period (550.5 cm). During the 2003 drought, the maximum water level (119.0 cm) was below the 5th percentile of the historical period (130.5 cm).

### Community response

Vascular plants were collected in 12 years between 1998 and 2014 (Supplementary Table S2), during which time a total of 260 plant species were recorded. Over 6 years between 1998 and 2006, 168,053 individuals of Carabid beetles, belonging to 181 species, and a total of 72,267 molluscs, representing 54 species, were collected.

**Figure 4:**
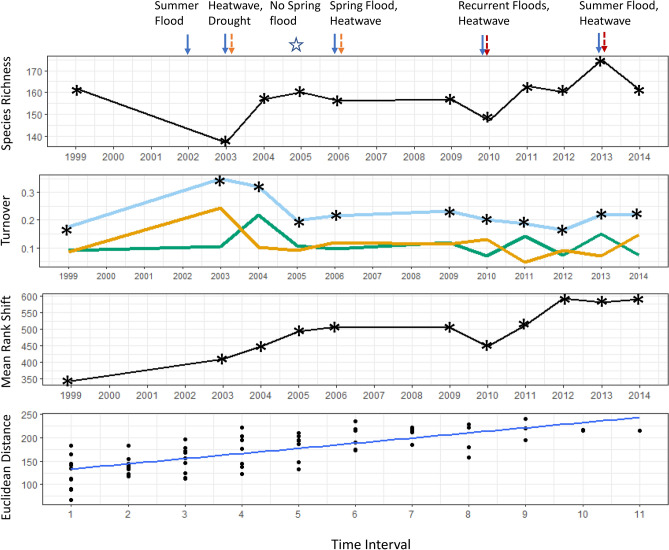
(**a**) Plant species richness, (**b**) turnover (total = light blue, appearances = green, disappearances = orange), (**c**) mean rank shifts and (**d**) community change over time. Arrows indicate years in which extreme events occurred, asterisks indicate sampling years. The first sampling year (1998) is not shown because all indices other than species richness are calculated as the difference between successive years. Species richness was 161 in 1998. Samples were taken in autumn 2002 but are excluded here as spring samples were not collected. Turnover for 2003 therefore refers to the turnover from 1999 to 2003. The slope of the line in the Euclidean distance panel indicates the rate and direction of community change. Euclidean distance is calculated for pair-wise samples across the time series, which is regressed against the time lag interval (see “Materials and methods” section for more detail). Note that the axis in this panel relates to the time interval between sampling periods (n = 11), not the sampling year.

**Figure 5:**
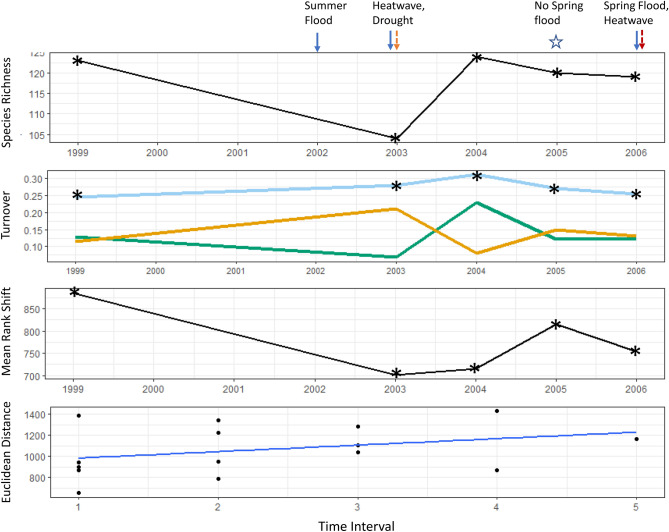
(**a**) Carabid species richness, (**b**) turnover (total = light blue, appearances = green, disappearances = orange), (**c**) mean rank shifts and (**d**) community change over time. Arrows indicate years in which extreme events occurred, asterisks indicate sampling years. The first sampling year (1998) is not shown because all indices other than species richness are calculated as the difference between successive years. Species richness was 121 in 1998. Samples were not taken between 1999 and 2003, so turnover for 2003 refers to the turnover from 1999 to 2003. The slope of the line in the Euclidean distance panel indicates the rate and direction of community change. Euclidean distance is calculated for pair-wise samples across the time series, which is regressed against the time lag interval (see “Materials and methods” section for more detail). Note that the axis in this panel relates to the time interval between sampling periods (n = 5), not the sampling year.

**Figure 6:**
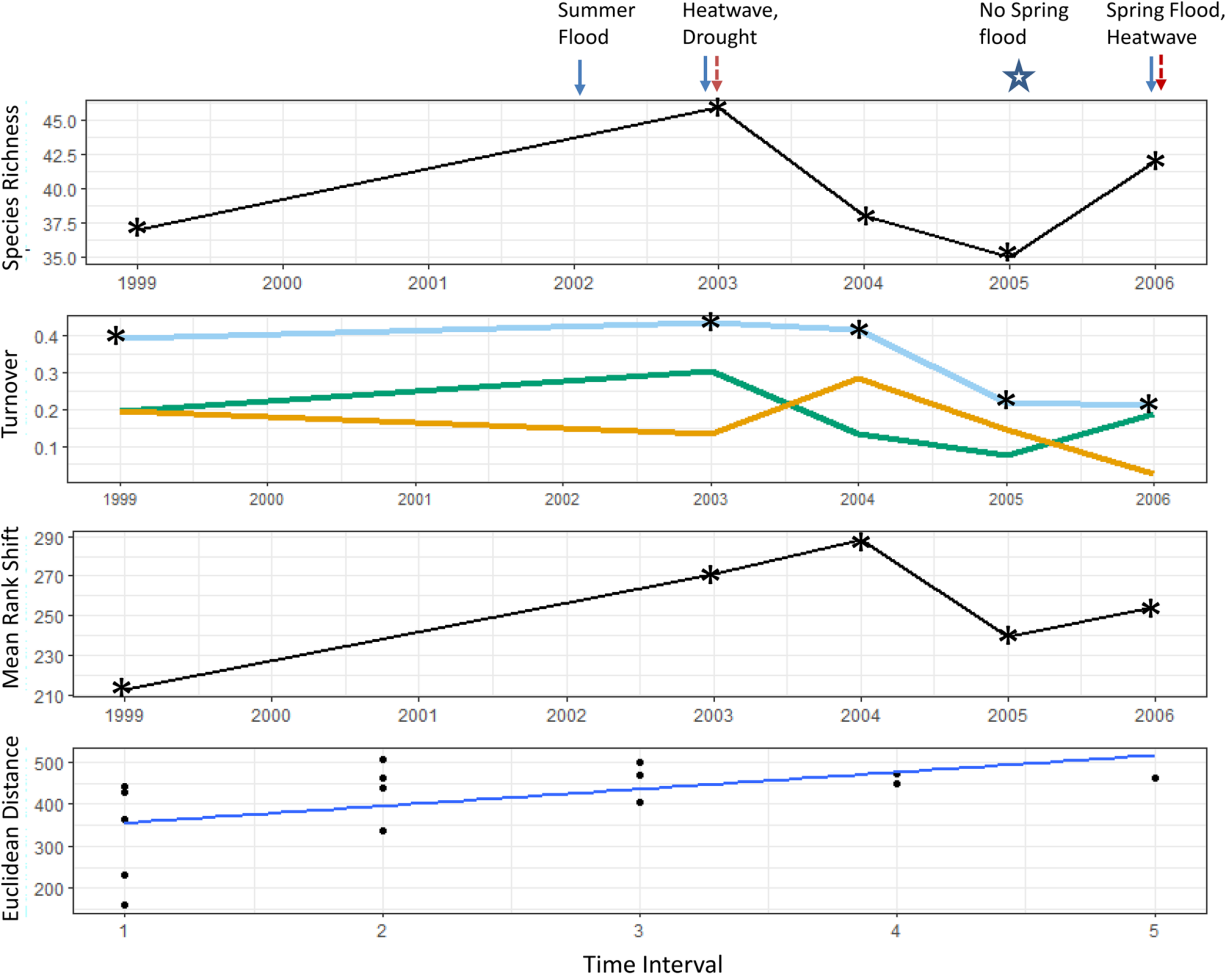
(**a**) Mollusc species richness, (**b**) turnover (total = light blue, appearances = green, disappearances = orange), (**c**) mean rank shifts and (**d**) community change over time. Arrows indicate years in which extreme events occurred, asterisks indicate sampling years. The first sampling year (1998) is not shown because all indices other than species richness are calculated as the difference between successive years. Species richness was 37 in 1998. Samples were taken in autumn 2002 but are excluded here as corresponding spring samples are missing. Turnover for 2003 therefore refers to the turnover from 1999 to 2003. The slope of the line in the Euclidean distance panel indicates the rate and direction of community change. Euclidean distance is calculated for pair-wise samples across the time series, which is regressed against the time lag interval (see “Materials and methods” section for more detail). Note that the axis in this panel relates to the time interval between sampling periods (n = 5), not the sampling year.

Species richness and turnover varied over the sampling period for each biological community (Figs. 4, 5, 6). However, in all communities, composition did not substantially differ between the start and end of the sampling period (indicated by the rate of community change).

Plant species richness was at its lowest in 2003 (Fig. 4), the year following the extreme flood and summer heatwave and dry period. The next year, it returned to levels similar to those found in 1999, after which time it remained relatively stable until 2010, when it declined (coincident with the recurrent floods and summer heatwave). Between 2010 and 2013, richness increased and then declined following the 2013 flood and heatwave. Total turnover in the vegetation community exceeded 17% in every year and reached 35% in 2003 (turnover since 1999). Close to 25% of plant species disappeared in this year and approximately 20% appeared the following year. Total turnover was relatively stable after 2005, but this was because the number of disappearances was similar to the number of appearances. Increased plant species re-ordering occurred from 2003 to 2005, following the 2003 heatwave and drought. This is reflected in the large number of species disappearances in 2003. In contrast, species re-ordering did not increase following the heatwave and summer flood of 2013. In this year, species disappearances increased and appearances declined, but there was little change in mean rank shift. The greatest mean rank shift occurred between 2010 and 2012, when several species appeared in 2011 and then disappeared the following year.

The carabid community had the highest species reordering over time compared with the other two taxonomic groups, and the highest rate of community change over the period (Fig. 5). Carabid species richness decreased substantially between 1999 and 2003 but returned to the initial richness between 2003 and 2004 (following the heatwave and dry period), when 20 species reappeared. Total turnover was high (25%) between all sampling periods, possibly due to low detection probability of the rarer and less mobile species. Between 2003 and 2004, 10% of carabid species disappeared, but were replaced by a similar number of new species. Even in consecutive years with similar species richness (e.g. 2005–2006), the identity of the composite species differed by 25%. The degree of species re-ordering declined over the four years between the 1999 and 2003 sampling periods, but without the 2002 sample it cannot be determined whether the change occurred gradually over the years from 1999 to 2003, or suddenly. Nevertheless, the 2002 summer flood was not immediately followed by increased species re-ordering, which only increased markedly between 2004 and 2005 (when no spring flood occurred).

The molluscs showed the highest total turnover of the three taxonomic groups (Fig. 6). Total turnover was up to 40% between most sampling years, compared to 25% in carabids and plants (20% in most years, highest turnover of 30% in 2003). The greatest decline (~ 20%) in mollusc species richness and highest turnover occurred between 2003 and 2004 following the heatwave and drought. Species richness increased again after the 2006 flood and heatwave. The degree of species re-ordering increased between 1999 and 2004 sampling periods, and decreased markedly between 2004 and 2005, when no spring flood occurred. This is the opposite pattern to that shown in the carabid community.

There were substantial shifts in species dominance in all communities over the sampling period (rank clocks presented in Supplementary Figures S1–S8). Additionally, responses varied at different sites and within plots with different inundation regimes. Community composition varied across the sites, and several changes were site-specific.

## Discussion

Formal attribution, as defined by the IPCC, requires three steps to be fulfilled: extreme event attribution, impact detection, and impact attribution (Fig. 1). At each step, three essential components are required^22^. First, the relationship between cause and effect must be demonstrated. Then the detected change must be shown to be inconsistent with changes due to alternative possible drivers. And finally, a quantification of the strength of the attribution statement is required to acknowledge the uncertainty and limitations of the available data and analyses*. *The challenges associated with these steps are clearly illustrated in the floodplain grassland community of the River Elbe.

Recent attribution studies have evaluated the extent to which human-induced climate change has affected extreme heatwaves, drought and floods in the Elbe River region. Stott et al.^38^ estimated the likelihood of a heatwave of the magnitude of the 2003 European one was at least doubled under human induced climate change (confidence level > 90%). Similarly, anthropogenic forcing was found to have played a substantial role in the hot, dry summer of 2013, both in terms of the high temperatures observed and the northward shift of the North Atlantic summer storm track which led to reduced rainfall over western Europe^39^. In contrast, a large simulation ensemble and observation-based analysis concluded that climate change had not made the extreme rainfall of 2013 in the upper Danube and Elbe basins more likely^40^. The attribution of rainfall events is substantially more difficult than temperature events because event attribution relies on the model’s ability to simulate the climate conditions generating the weather event. This remains challenging for rainfall, which is naturally highly variable, and generated by processes that are not captured well at the scale of current-generation climate models^41,42^. Flood time series are similarly highly variable in response to natural variability and factors such as urbanization, deforestation and dike construction. These factors occur simultaneously across a catchment and often interact at multiple temporal and spatial scales, limiting attribution of extreme floods to climate change^42,43^.

Understanding natural modes of variability plays a crucial role in attribution studies, particularly for rainfall and associated flooding events. For example, the apparent increase in intense rainfall that we identified in the current period, particularly in summer, could have been caused, in part or fully, by natural variability. The 20-year time periods used here are sufficient to capture interannual drivers of variability such as the El Nino–Southern Oscillation and decadal modes such as the North Atlantic Oscillation and the Pacific Decadal Oscillation. However, a significantly longer time period would be needed to capture multidecadal patterns such as the AMO, which was predominantly negative during the historical period and positive during the recent period^44^. The lack of long-term observations means there are few studies of the influence of the AMO on the study region, although increased summer rainfall has been associated with a positive AMO in modelling over North Western Europe^45^.

It was possible to detect a community response to the extreme events. The composition and abundance of all taxa displayed considerable inter-annual variability, but changes in community turnover, species abundance and dominance were detected in years following the extreme events as previously shown by^31^. Decreased carabid species richness was found in years following drought and heatwave events, while plant species richness increased or remained stable in years after heatwaves and/or drought. Extreme floods reduced the species richness of plants and beetles in the short term. In contrast, and consistent with other studies, molluscs showed higher species richness following flooding and lower richness after heatwave and drought years^46–48^. Many of the changes detected were consistent with expectations based on understanding of life cycle biology, such as timing of reproduction and traits enabling inundation and drought tolerance (e.g. molluscs with diaphragms or lids that close shells), although not all changes had simple explanations (described in more detail in Supplementary Information).

While “detection” does not depend on an explanation of the causes of the observed change^20^, it does require a demonstration that the likelihood of the response is significantly different from that due to natural variability. This is challenging with biological data, for several reasons. First, extreme events occur infrequently and are difficult to predict, so it is rare to have baseline data to characterise the community prior to an event. Second, biological data are generally highly variable in space and time. As illustrated in the floodplain community, species abundance, composition and dominance commonly vary over time, with responses differing both within and between sites. In some cases, the same species responded differently under different conditions.

Further, a community response to an extreme event may be sudden or gradual, periodic or episodic, and the effects may be short-term or permanent. Without continuous observations, it is impossible to determine whether changes in community indices occurred gradually over the years from 1999 to 2003, or suddenly in response to the 2002 extreme flooding event. It is difficult to quantify the extent to which the community is altered permanently by one extreme event. In the dynamic floodplain system there may be some capacity to return to a previous state, since many organisms are adapted to cope with regular flooding events. However, species interactions and feedbacks could lead to lagged responses due to changes in resource availability or competitive interactions^49^.

Additionally, ecosystem responses may not always occur immediately after a single weather event, but in response to the long-term stress of the changing climate, in combination with extreme weather events^9^ (the ‘press and pulse’ framework^17^). Mean temperature has increased in the River Elbe region over recent decades, so any community response may be influenced by this change in the background ‘press’, as well as the magnitude, duration, frequency and timing of the extreme ‘pulse’ events.

The severity of flood impact will also be affected by changes in timing in relation to the traits and phenological stages of species within the community^50^. Annual spring floods resulting from snowmelt represent natural variability to which the community is adapted. This is supported by the fact that all taxa declined in species richness following the year in which the spring flood did not occur. However, the summer floods of 2002 and 2013 were extreme in terms of severity, duration, and timing. Floods occurring in summer were associated with reduced species richness in carabids and plants. Carabids exhibit adaptations to flood such as autumn emigration, hibernation as adults or physiological adaptations such as low physiological activity or higher submergence resistance in low temperatures. These adaptations enable survival through the usual winter and spring floods, but do not confer resilience to summer floods, which occur when many species are in sensitive larval or pupal phases^31^.

Differences in traits across taxa mean that different responses will be shown by different taxonomic groups^51^. The ability to detect a response will therefore depend on what “community” is of interest. Here we found, for example, the pattern of species re-ordering over time was in the opposite direction in the carabids and molluscs after the 2002 flood and 2003 heatwave. Both aquatic and terrestrial molluscs are well adapted to floods, as even land snails can survive in water provided the water is oxygenated and not too warm. In contrast, carabid beetles range in their ability to survive inundation and dispersal ability is important for recolonising after floods^52^.

Multiple events also complicate detection^53^. Here, for example, the low mean monthly precipitation recorded in 2003 fell within the lower 25th percentile, so does not represent a climatological extreme in isolation. However, at the same time, extreme maximum temperatures were recorded for extended periods (Table 1) and water levels were significantly lower than the long-term mean (Supplementary Figure S1), with the maximum water level below the 5th percentile of the historical period (Fig. 3d). A strong biological response in all taxonomic groups was associated with the year 2003, in which extended heatwaves coincided with low water levels. However, plant data from 2010 suggest that a similar response as that found in 2003 (increased species richness and decreased turnover) is also associated with recurrent flooding in combination with heatwaves. The mechanisms driving the response are obviously quite different, associated with the added nutrients provided by the fine sediments carried by floods^54^.

In the current case, not only were the impacts of droughts and heatwaves superimposed on the impacts of floods (natural and extreme), but these events are likely to act in opposing directions. In the short term, floods act to homogenise the habitat and provide nutrients, while drought and heatwaves are more likely to increase heterogeneity across microhabitats with differing elevations and exposure to water^50^. Over longer timescales, however, increased homogeneity could be expected as the habitat dries out in the absence of regular flooding events. The impact of drought and heatwaves on floodplain communities is likely to be greater than that of floods, given the high proportion of aquatic and inundation-dependent and tolerant species.

Attribution in the climate system relies on the ability to quantitatively model the system^55^. The mismatch in temporal and spatial resolution between available biological data and climate observations and models^42^ limits the ability to apply statistical analyses and develop models, and reduces the chances that responses at fine spatial resolution will be successfully attributed to climate change. This is compounded by the high natural variability in the climate variables of interest, in addition to the variability in biological communities, as discussed above. Each extreme event is essentially not replicable^16^, and even where multi-event responses are available, each event has specific characteristics. Attempts to link biological responses to climate change are therefore likely only to be possible at continental to global scales, or over the timescale of decades^56^.

Interactions and feedbacks are important structuring factors in natural systems. Extreme events can alter species interactions by reducing populations of common species, allowing another species to increase in population sufficiently to prevent the dominant species re-establishing. In many cases, quantification of such interactions remains problematic, and cause and effect cannot be inferred from correlations between observations and events.

Separation of drivers is a key element of formal detection and attribution analysis^21^. Biodiversity responses, however, are likely to be driven by multiple factors, acting on a range of timescales. Hydrologic conditions, land use and management, for example, are important drivers of vegetation and invertebrate floodplain communities (e.g.^57–60^). In some cases, such as the impact of mowing, riverbed erosion or water extraction, the non-climate driver is easily identified. However, many land use changes develop over decades to centuries, and so would more likely have a long-term effect on biodiversity. Multiple-driver attribution would require the role of such non-climatic drivers to be accounted for and shown to be inconsistent with the observed community response.

The case of the floodplain community suggests that the formal joint attribution of community responses to extreme events caused by climate change will rarely be possible. The detection of responses to extreme events, however, is feasible, and important to improve understanding of the connections between climate, extreme weather events and biodiversity. Such knowledge is essential to inform conservation management attempts to mitigate the impacts of extreme events or ongoing climate change.

Monitoring is essential, ideally before and particularly after an extreme event, to improve our knowledge of the connections among climate, weather and biodiversity. Baseline data needs to be long-term and spatially extensive, due to the highly variable nature of biological data^61^. More intensive temporal monitoring is essential to better understand patterns and drivers of natural variability so that extreme responses may be identified. Improved spatial replication would increase the likelihood of having biological data from areas that did not experience the extreme event and could also enable other important, non-climatic, drivers to be identified^16^. Better spatial monitoring is also necessary to predict a response to extreme events in ecosystems other than those with long-term observations.

Long-term monitoring should be designed within a sound hypothesis testing framework^16^ to encourage a more thorough consideration of the important (and possibly interacting) drivers and their potential effects on biological communities and the mechanisms driving change. Although some taxa are more difficult and expensive to sample, it is important that the best taxon to identify a community response is monitored. While plants are the cheapest and easiest taxon to monitor, they might not be the most appropriate group, as differences in biology across taxonomic groups are likely to lead to a range of responses^56^.

To strengthen our understanding of impacts and responses, biological monitoring should be complemented with evidence from observations, remote sensing, experimental data, models and ecological theory. Experiments to identify mechanisms driving a response can support observational studies and establish causal relationships^11,16,62^. For example, Rothenbucher and Schaefer^63^ used exclosure plots on the Lower Oder floodplain after the 2002 flood to identify species responses in relation to inundation tolerance and immigration. Such experimental information, combined with observations over time, could contribute to greater understanding of how extreme events affect the distribution of species and the structure of communities.

Mesocosm experiments are particularly appropriate for testing the impacts of extreme events on aquatic and riparian invertebrate communities. Experimental manipulations of temperature and moisture can be used to test hypotheses generated by observations of community responses and determine cause and effect^64^. An additional advantage of mesocosm experiments is the ability to incorporate the effect of carbon dioxide on species responses, an important component that is frequently ignored in observational studies of global change. While short-term experiments can provide important biological knowledge, longer-term mesocosm experiments are essential to identify ongoing impacts of extreme events on the structure and function of communities, including potential lag effects, feedbacks and interactions^65^.

Meta-analyses to combine results from studies of different single extreme events are needed to consolidate observations and identify trends, similarities, divergences and exceptions. Such analyses will be more informative if studies report similar aspects in a comparable way. For instance, the magnitude of the extreme event should be defined and robust estimates of the magnitude of ecological responses and other drivers reported^1,16^. Global and regional trends are more likely to be identified through such syntheses.

Reanalysis products based on climate observations could help link weather patterns associated with an extreme event and an observed biological response. Such products are now available at resolutions fine enough to capture processes at biologically relevant scales. For example, the NWP model COSMO regional reanalysis data sets^66,67^ provide hourly atmospheric data for Europe with a resolution of 6 km for the years 1995–present. The application of reanalysis products, seasonal forecasts and high-resolution projections will strengthen the link between biological and climate knowledge.

Central Germany, in common with many regions of the world, has experienced several extreme weather events over recent decades, in addition to gradual background warming. There is increasing interest in attributing biological responses to extreme events and climate change, but there are many challenges that limit our ability to achieve formal, quantified joint attribution. Nevertheless, it is important that we detect responses and improve our understanding of the mechanisms behind change to inform conservation management and restoration. This is particularly important as the incidence of extreme events is projected to increase in the future.

## Materials and methods

### Study regions and sites

Floodplain plant, mollusc and carabid beetle communities were sampled from the Middle Elbe River, Germany between 1998 and 2014 (plants 1998–2014, molluscs and beetles 1998–2006). Data were collected from three sites (Fig. 2) using standardised sampling methods following a stratified randomized sampling design (details in^68^). Within each site there were three plot types: (1) flood-channels, (2) wet grassland; and (3) mesophilous grassland. Due to differing site morphology these plot types are exposed to different frequency and duration of flooding and therefore represent a gradient in disturbance frequency^68^.

### Climate data

Mean monthly summer temperature (air and water), mean monthly rainfall and mean annual water level were calculated for the historical period (1961–1990) and compared to the values for the recent period (1996–2015), which includes the years in which biological sampling was carried out (1998–2014). The periods are not continuous, but represent a baseline, or historical period, and the current period. Mean climate data from the nearest weather stations with data covering the period 1961–2015 were used to indicate local conditions. Data were provided by the German Meteorological Service [Deutscher Wetterdienst (DWD)]. Water level data were provided by the Federal Institute of Hydrology (BfG), from the Aken Elbe 274.7 km gauge [Federal Waterways and Shipping Administration (WSV)] (Supplementary Information Table S1).

We use twenty-year time periods, as is common in climatological studies, to incorporate decadal variability due to the influence of large-scale climate drivers. The 20-year length of the time periods is sufficient to cover interannual drivers of variability such as the El Nino–Southern Oscillation and decadal modes such as the North Atlantic Oscillation and the Pacific Decadal Oscillation. The time periods cover both positive and negative phases of the North Atlantic Oscillation, the Atlantic Multidecadal Oscillation (AMO) and El Niño Southern Oscillation (ENSO) (NOAA Center for Climate Prediction. https://www.cpc.noaa.gov/, Retrieved 23rd May 2017). However, the length may not be sufficient to capture fully multidecadal patterns such as the AMO, which was predominantly negative during the first period and positive during the second period^44^.

We use probability density functions (pdfs) to describe shifts in mean and extreme conditions. This approach gives an indication of the spread of the observations over the period of interest and enables extremes to be described relative to the local levels of climate variability. Since it is this local variability that organisms have adapted to, the magnitude of the deviation from the mean has greatest biological impact^17^. We therefore follow the common convention^3,69,70^ of defining extreme events as events that fall within the 10th, 90th or 99th percentile of the probability density function (pdf) based on historical observations.

Time series analysis was used to assess and visualize the main components of variability (the trend, the seasonal and the random components) in monthly air temperature, monthly precipitation and monthly water level (the variables with sufficiently long timeseries for analysis). Since the primary focus of this study is to detect biological responses to extreme events, rather than to long-term climate trends, these analyses are presented in the Supplementary Material (Supplementary Figures S2–S4).

Each extreme weather event during the period 1995–2014 was compared to the historical climate observations to determine whether they represented extreme climatological events (as defined by the IPCC: > 90th or 99th percentile^70^). We include 1995 to consider the potential impacts of events occurring just prior to the sampling period. The number and duration of summer heat events in each year, based on maximum summer daily values, was calculated as the number of consecutive days above the historical 99th percentile summer temperature^71^. Flood and drought events were compared based on maximum water level during each flood or drought event.

### Community data and analysis

We use field data collected in the floodplains of the Middle Elbe River, Central Germany, using standardised sampling methods across well replicated plots within sites (details in^68^). The abundance of vascular plants, molluscs and carabid beetles at three floodplain grassland sites [Steckby (n = 36), Wörlitz (n = 12) and Sandau (n = 12); Fig. 3] were assessed in spring and autumn of each sampling year (Supplementary Information, Supplementary Table S2). Here we present annual sums of abundance. No sampling took place in 2000–2002. Sampling restarted following the extreme flood of August 2002.

The biological dynamics within the relatively short seasonal sampling periods are driven more by differing hydrological and weather conditions (inundation, ground-water level and fluctuations, dryness, and management before and during the sampling periods) than by the taxa´s biological phenomena. In Central Europe seasons are very pronounced and their starting date can differ by 2–3 weeks across years. As a result, the sampling dates varied slightly across the years to minimise differences in species composition or abundance in each of the taxa. For example, sampling time in spring was determined by the beginning of flowering (particularly important for identification of geophytes). In the flood channels, sampling was delayed at times by the need to wait for flooding to subside. In autumn, sampling was carried out once fresh growth had occurred following the long dry periods in summer. Slightly different time periods therefore lead to greater comparability of the samples, as they capture the effects of interannual variability in weather and hydrology.

There are many metrics available to measure biodiversity, from univariate indices describing species richness, diversity and evenness, to multivariate descriptions of community composition. We use three types of temporal diversity indices: (1) species turnover, (2) mean rank shifts and (3) the rate of compositional change in community composition over time, as described in Hallett et al.^72^.

i) Species turnover is the proportion of species that differ between time points, calculated as:
  $${\text{Total turnover }} = {\text{Species gained}} + {\text{Species lost}}$$
  Total species observed in both time points.
ii) The mean rank shift (MRS) quantifies relative changes in species rank abundances, which could indicate reshuffling of species within the same community type or successional change from one type to another. It is calculated as:
  $${\text{MRS }} = \sum\limits_{i = 1}^{N} {\left( {|{\text{R}}_{{{\text{i}},{\text{t}} + {1}}} - {\text{R}}_{{{\text{i}},{\text{t}}}} |} \right){\text{/N}}} ,$$
  where N is the number of species in common in both time points, t is the time point and R_i,t_ is the relative rank of species i at time t.
iii) Rate and direction of compositional change in the community over time.

We applied the time-lag analysis of Collins et al.^73^ to measure the rate of change in community composition over time. This approach is a community level extension of autocorrelation analysis, but Euclidean distance is used to measure similarity of pair-wise community samples at increasing time lags instead of the correlation coefficient. It can therefore identify trends in time-series that are not long enough to be subjected to more traditional forms of time-series analysis.

The Euclidean distance is calculated for pair-wise samples across the time series. For example, the mollusc and beetle datasets, with five sampling periods, will have distance values for four one-interval time lags (e.g. t1 vs t2, t2 vs t3 etc.), three two-interval time lags (e.g. t1 vs t3, t2 vs t4 etc.) and so on. The distance values are then regressed against the time lag interval, the slope of which shows the rate of community change^72^. Where sampling years are missing, the Euclidean distance spans the missing years.

In addition, we use rank clocks to visualize changes in the rank order of abundance of the dominant species within each taxonomic group over time^74^. The four most abundant species in each year from each plot type and site (see Supplementary Figures S6–S8) are presented separately, because species composition differed both within and between each site.

All analyses were done using the R package ‘codyn’^72,75^.

## Supplementary information

Supplementary file1

## Data Availability

The datasets analysed in the current study are not yet publicly available due to the ongoing nature of the research with higher degree research students but are available from the corresponding author on reasonable request.
