# Supplementary material for: Biological responses to extreme weather events are detectable but difficult to formally attribute to anthropogenic climate change
Source: Sci Rep. 2020 Aug 21;10:14067. doi: 10.1038/s41598-020-70901-6 (PMC7442817; doi:10.1038/s41598-020-70901-6)
Supplement: Supplementary file 1 — Supplementary file1 [file 41598_2020_70901_MOESM1_ESM.pdf]

Supplementary Information

Biological responses to extreme weather events are detectable but difficult to formally attribute to anthropogenic climate change

Harris, R.M.B.*[1,2,3], Loeffler, F.[1], Rumm, A. [4], Fischer, C. [1, 5],, Horchler, P. [5], Scholz, M.[1], Foeckler, F. [4], Henle, K.[1]

[1] Department of Conservation Biology, Helmholtz Centre for Environmental Research – UFZ, Leipzig

[2] Antarctic Climate & Ecosystems Cooperative Research Centre, University of Tasmania, Hobart

[3] Geography and Spatial Sciences, University of Tasmania, Hobart

[4] ÖKON GmbH, Hohenfelser Str. 4, Rohrbach, 93183 Kallmünz, Germany

[5] Institute for Geosciences, Friedrich-Schiller-University Jena, Burgweg 11, 07749 Jena, Germany

[6] Department Vegetation Studies, Landscape Management, German Federal Institute of Hydrology (BfG), Am Mainzer Tor 1, 56068 Koblenz, Germany

[7] German Centre for Integrative Biodiversity Research (iDiv) Halle-Jena-Leipzig, Deutscher Platz 5e, 04103 Leipzig, Germany.

* rmharris@utas.edu.au, +61 (0)3 6226 2920

Supplementary methods include:

1. The location of the weather stations from which observations were taken from 1961-2015;
2. Sampling dates for each taxon;
3. The River Elbe water level during event years 2002, 2003, 2006, 2010 and 2013 in relation to the mean water level between 1980-2006.
4. A description of the time series analysis used to assess climate variability over the period 1961-2015

Supplementary results show:

1. The seasonal summary of precipitation changes between the baseline period (1961-1991) and the sampling period (1996-2015);
2. time series decomposition for monthly air temperature, precipitation and water level from 1961 to 2015, and
3. Rank clocks showing dominance structure of the three communities (Plants, Carabid beetles and molluscs) at each sampling site (Sandau, Steckby and Wörlitz).

Supplementary Methods

Supplementary Table S1: Weather stations closest to the study sites with observations from 1961-2015.

Variable	Weather Station	Latitude, Longitude	Distance to closest site
Air temperature	Bernburg/Saale (Nord)<br>Wittenberg	51° 49' 26.39" N, 11° 42' 18" E<br>51° 53' 20.25" N, 12° 38' 41.24" E	Steckby, 38km<br>Wörlitz, 23 km
Precipitation	Aken/Elbe<br>Wittenberg	51° 51' 17.99" N, 12° 2' 34.79" E<br>51° 53' 20.25" N, 12° 38' 41.24" E	Steckby 9km<br>Wörlitz 23 km
Water temperature	Bunthaus, Elbe<br>Grauerort<br>Schnakenburg<br>Seemannshöft	53° 27' 42.34" N, 10° 3' 51.75" E<br>53° 40' 40.46" N, 09° 29' 42.01" E<br>53° 02' 17.93" N, 11° 34' 11.68" E<br>53° 32' 24.77" N, 09° 53' 10.35" E	Sandau 51km
Water level	Aken/Elbe<br>Wittenberg	51° 51' 17.99" N, 12° 2' 34.79" E<br>51° 53' 20.25" N, 12° 38' 41.24" E	Steckby 9km<br>Wörlitz 23 km

The probability distribution functions of the variables were calculated from the weather stations as follows: a) mean monthly summer air temperature (mean of Bernburg/Saale (Nord) and Wittenberg); b) annual observed daily water temperatures (mean of Bunthaus, Grauerort, Schnakeburg, Seemannshoft); c) mean monthly precipitation (Bernburg/Saale (Nord) and Wittenberg); d) annual water level (mean of Aken and Wittenberg).

Supplementary Table S2: Sampling dates for each taxon

Year	Vegetation		Carabidae		Mollusca	
	Spring	Autumn	Spring	Autumn	Spring	Autumn
1998	6th May-3rd June	28th Aug-29th Sept	27th Apr-7th Jun	2-30th Sept	27-15th May	23rd Sept-2nd Oct
1999	6th May-2nd June	7th Sept-12th Oct	27th Apr-15th Jun	23rd Aug-11th Oct	3-9th May	9-11th Oct
2003	21st May-31st Jul	7th Sep - 20th Oct	18th May-24th Jun	1st Sept-1st Oct	19-23rd May	8-12th Sept
2004	27th Apr-27th Jul	25th Aug-24th Sept	14th May-10th Jun	30th Aug-5th Oct	24-26th May	18-21st Oct
2005	29th Apr-15th Jul	30th Aug-14th Sept	13th Apr-8th Jun	6th Sept-6th Oct	27-29th Apr	6-8th Sept
2006	22nd May-20th Jul	22nd Aug-12th Sept	15th May-16th Jun	4th Sept-5th Oct	22-24th Apr	18-20th Sept
2009	9th May-30th Jul	25th Aug-17th Sept	-	-	-	-
2010	5th May - 28th Jul	6th Sept-13th Oct	-	-	-	-
2011	14th May-6th Jul	15th Aug-29th Sept	-	-	-	-
2012	2nd May-15th Jun	16th Aug-11th Sept	-	-	-	-
2013	16th May-17th June	26th Aug-22nd Oct	-	-	-	-
2014	8th May-25th Jun	19th Aug-2nd Sept	-	-	-	-

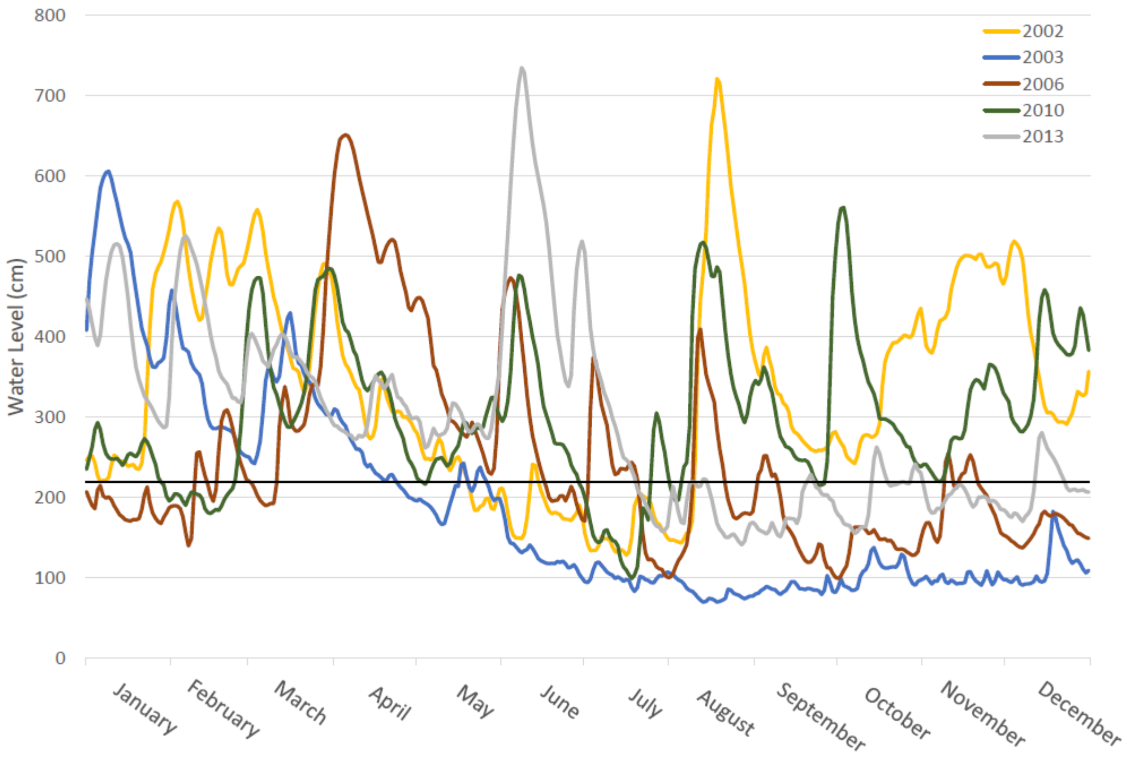

Supplementary Figure S1: River Elbe water level during event years 2002, 2003, 2006, 2010 and 2013. Horizontal line indicates mean water level between 1980-2006. Data provided by the Federal Institute of Hydrology (BfG), from the Aken Elbe 274.7km gauge (Federal Waterways and Shipping Administration (WSV), 2014).

Time series analysis to assess climate variability over the period 1961-2015

Time series analysis was used to assess and visualize the main components of variability - the trend, the seasonal and the random components. The variables with sufficiently long timeseries for analysis were monthly air temperature, monthly precipitation and monthly water level.

A seasonal decomposition of each time series using loess smoothing was applied using the stl function in the R stats package (Cleveland et al. 1990). In this algorithm, loess smoothing over a moving window is used to calculate the seasonal component of variability, which is removed from the overall level. The remainder is then smoothed to find the trend. The overall level is removed from the seasonal component and added to the trend component. The remainder is the residuals from the seasonal plus trend fit.

Supplementary Results

Decomposition of climate data into the seasonal, residual and trend components of variability

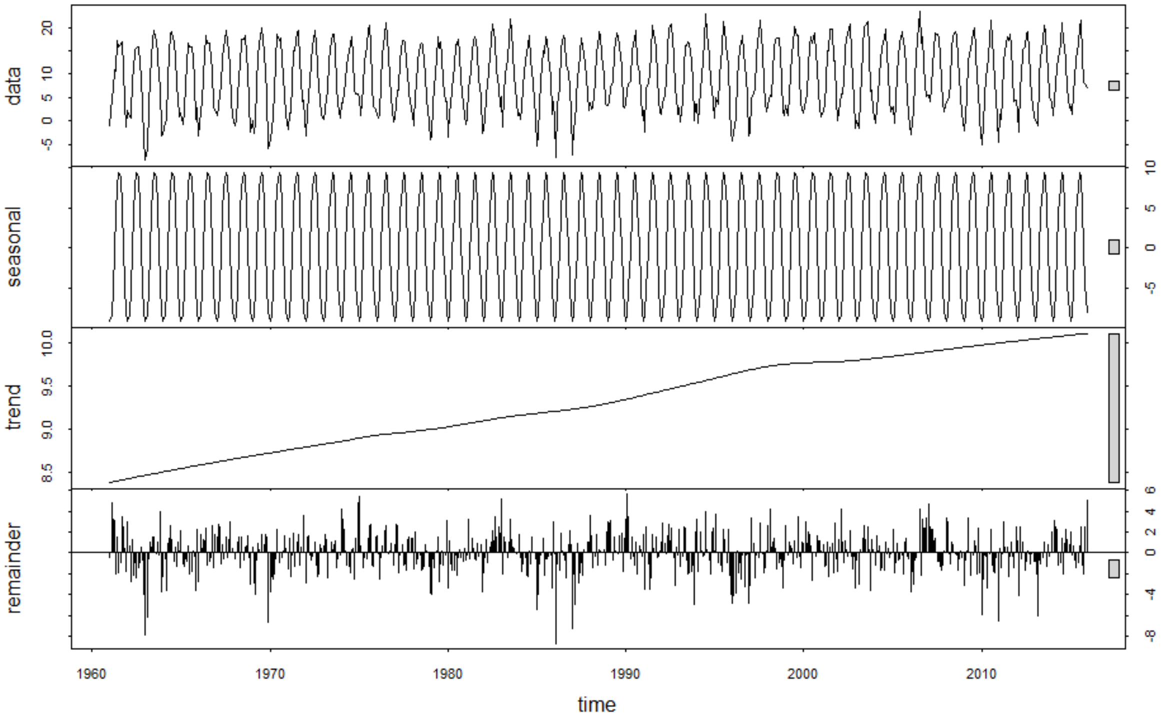

Supplementary Figure S2: Decomposition of mean monthly air temperature over the period 1961 to 2015. There is a warming trend of approximately 1.5°C after the seasonal variability is accounted for, although the trend is less than the monthly variation, indicated by the bar at the right-hand side of each graph showing the relative magnitude of each component.

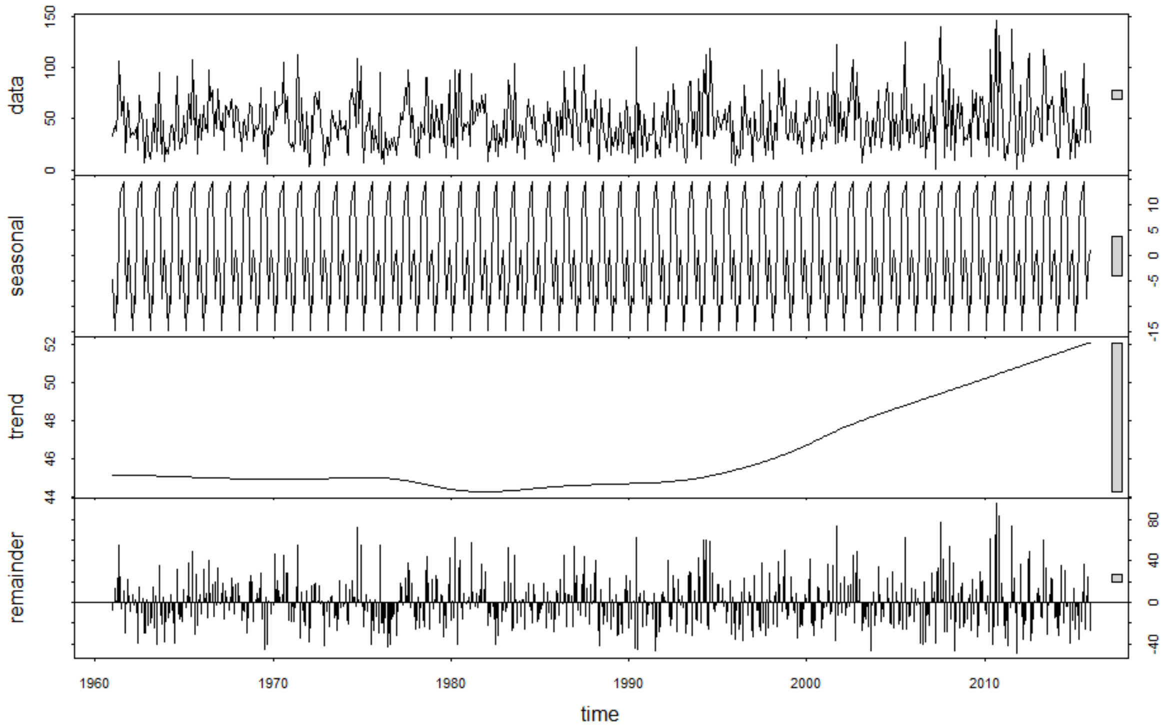

Supplementary Figure S3: Decomposition of mean monthly precipitation over the period 1961-to 2015. There is a slight positive trend in precipitation since 1998 once the seasonal variability is removed, although the trend in precipitation is less than the monthly variation, indicated by the bar at the right-hand side of each graph showing the relative magnitude of each component.

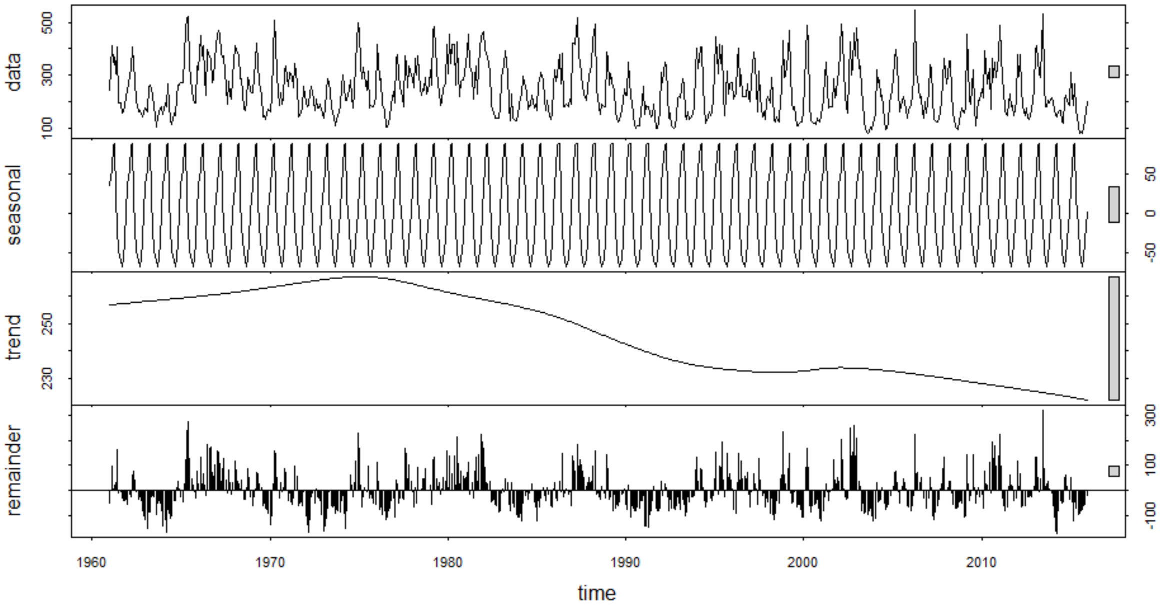

Supplementary Figure S4: Decomposition of mean monthly water level over the period 1961-to 2015. A slight decline in mean monthly water level since the 1980's is apparent once the seasonal variability is removed, although the trend in water level is less than the monthly variation, indicated by the bar at the right-hand side of each graph showing the relative magnitude of each component.

Seasonal summary of precipitation changes between the baseline period (1961-1991) and the sampling period (1996-2015)

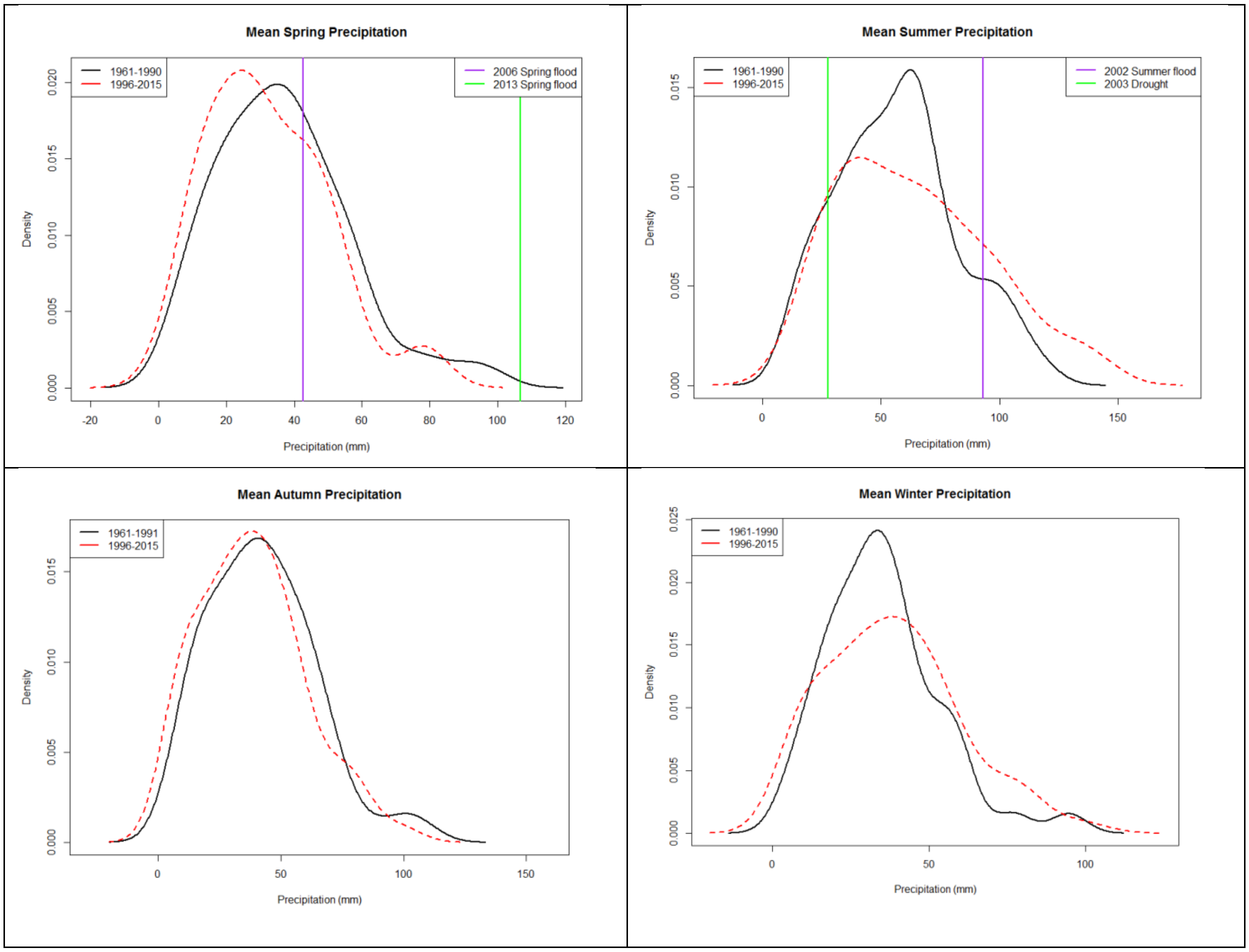

Supplementary Figure S5: Probability distribution function of mean seasonal precipitation (Bernburg/Saale (Nord) and Wittenberg), for the baseline period (1961-1991) and the sampling period (1996-2015). Maximum values for the two months preceding each extreme rainfall event are displayed as vertical lines. Spring (March, April, May); Summer (June, July, August); Autumn (October, November, December); Winter (January, February, March)

Community changes - Rank clocks showing dominance structure of the three communities at each sampling site

There were substantial shifts in species dominance in all communities over the sampling period (Supplementary Figures S6-S8). Responses varied at different sites and within plots with different inundation regimes. Community composition varied across the sites, and several changes were site-specific. The beetle community at the Steckby site, for example, showed very different patterns of abundance and richness compared to the other sites. The dominance structure at Wörlitz differed markedly from the other sites, and while similar carabid species occurred on both dry and wet plots, the changes in dominance over time differed widely. In both plots *Pterostichus melanarius* increased substantially between 2004 and 2006 to reach very high numbers. In the dry plots, there was little change to the abundance of other species, but in the wet plots, the dominance structure also changed, with *Nebria brevicollis* increasing after 2002, while *Poecilius versicolor* declined.

Many of the changes detected were consistent with expectations based on understanding of life cycle biology, such as timing of reproduction and traits enabling inundation and drought tolerance (e.g. molluscs with diaphragms or lids that close shells). For example, *Planorbis planorbis*, a mollusc species with high inundation tolerance usually characteristic of the wetter subplots (Foeckler et al. 2006), increased on the dry Sandau subplots after the 2002 flood, and declined following the 2003 heatwave and dry period (Figure S5). *Vallonia excentrica*, usually associated with drier conditions (Foeckler et al. 2006), increased on dry and moist subplots at the Wörlitz site after the 2003 heatwave and drought, and after 2005 when no spring flood occurred. Additionally, timing of reproduction in relation to the timing of each extreme event affected the response of species. For instance, the impact of the 2003 heatwave and drought was greater on *Vallonia pulchella*, whose reproductive period (May to August) occurred prior to the event, in

comparison to *Zonitoides nitidus,* which reproduces from September to December, after the heatwave occurred. Also consistent with known biological requirements, the Carabid species *Agonum micans,* usually associated with wet subplots and flood channels, decreased in the flood channel and wet Sandau sub-plots after heatwave/drought years and following 2005. In the plant community, the largest changes were in *Elymus repens.* At Sandau, this species declined and was replaced by *Urtica dioica,* a N-nutrient indicator species that would have benefitted from the increased nutrients provided by flood events. *E. repens* also declined at Wörlitz. In the flood channel subplots at Wörlitz, dominance shifted from *Carex acuta* to the hydrophyte *Persicaria hydropiper* over time. *P. hydropiper*, at this site and Sandau, declined following years with heatwave and drought, and increased following the recurring floods of 2010.

However, not all changes had simple explanations. For example, the carabid *Agonum afrum* increased to massive numbers (>300 on average per plot) on the wet subplots at Steckby in 2005, the year when there was no spring flood. Abundance then declined in 2006. Since this species is usually associated with flood channels and the wettest subplots, this is difficult to explain simply in terms of inundation tolerance.

a) Sandau

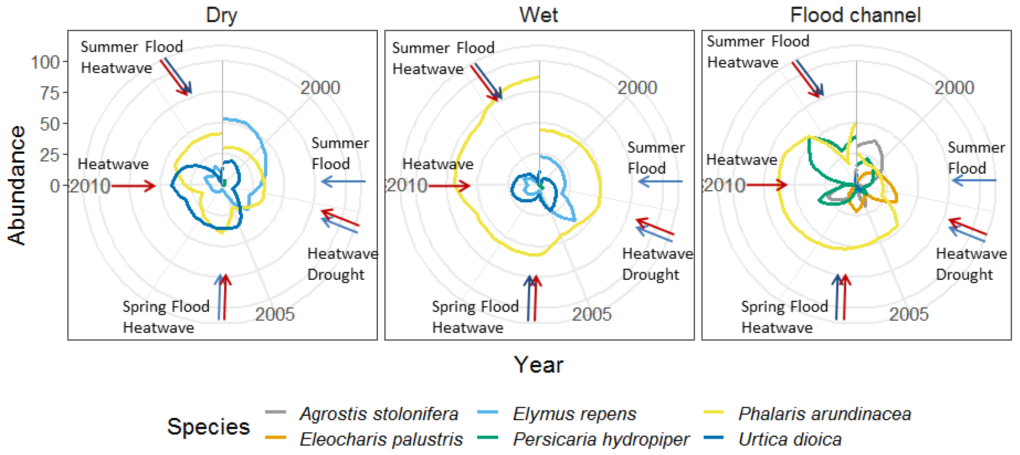

b) Steckby

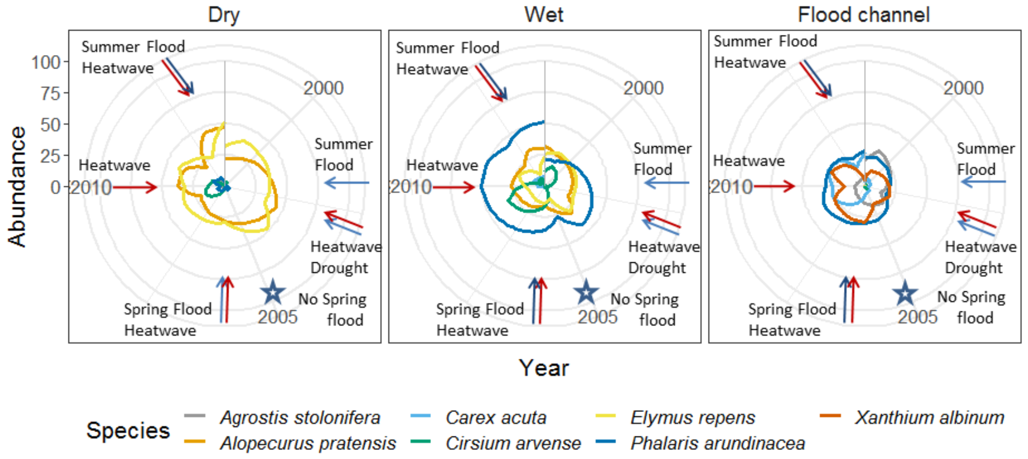

c) Wörlitz

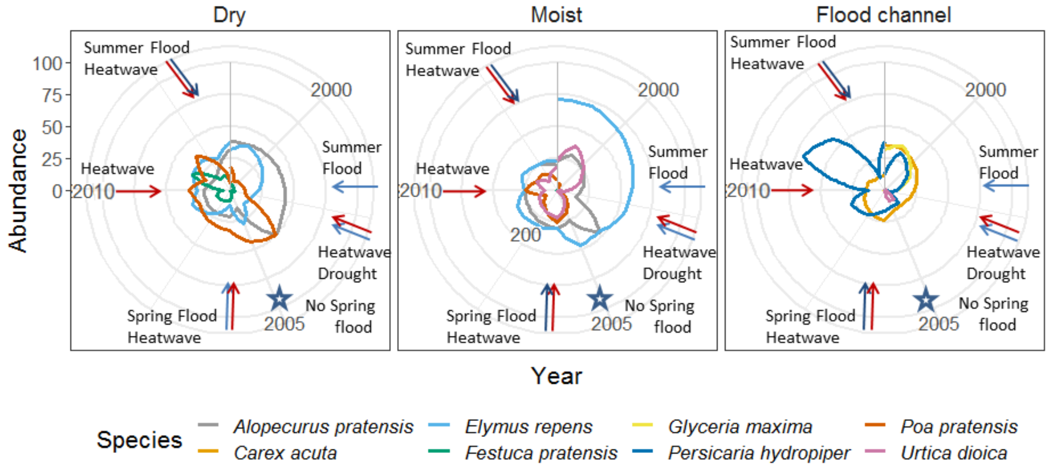

Supplementary Figure S6: Rank clocks showing dominance structure of the plant community in three plot types at Sandau, Steckby and Wörlitz. Abundance values are shown in the concentric rings.

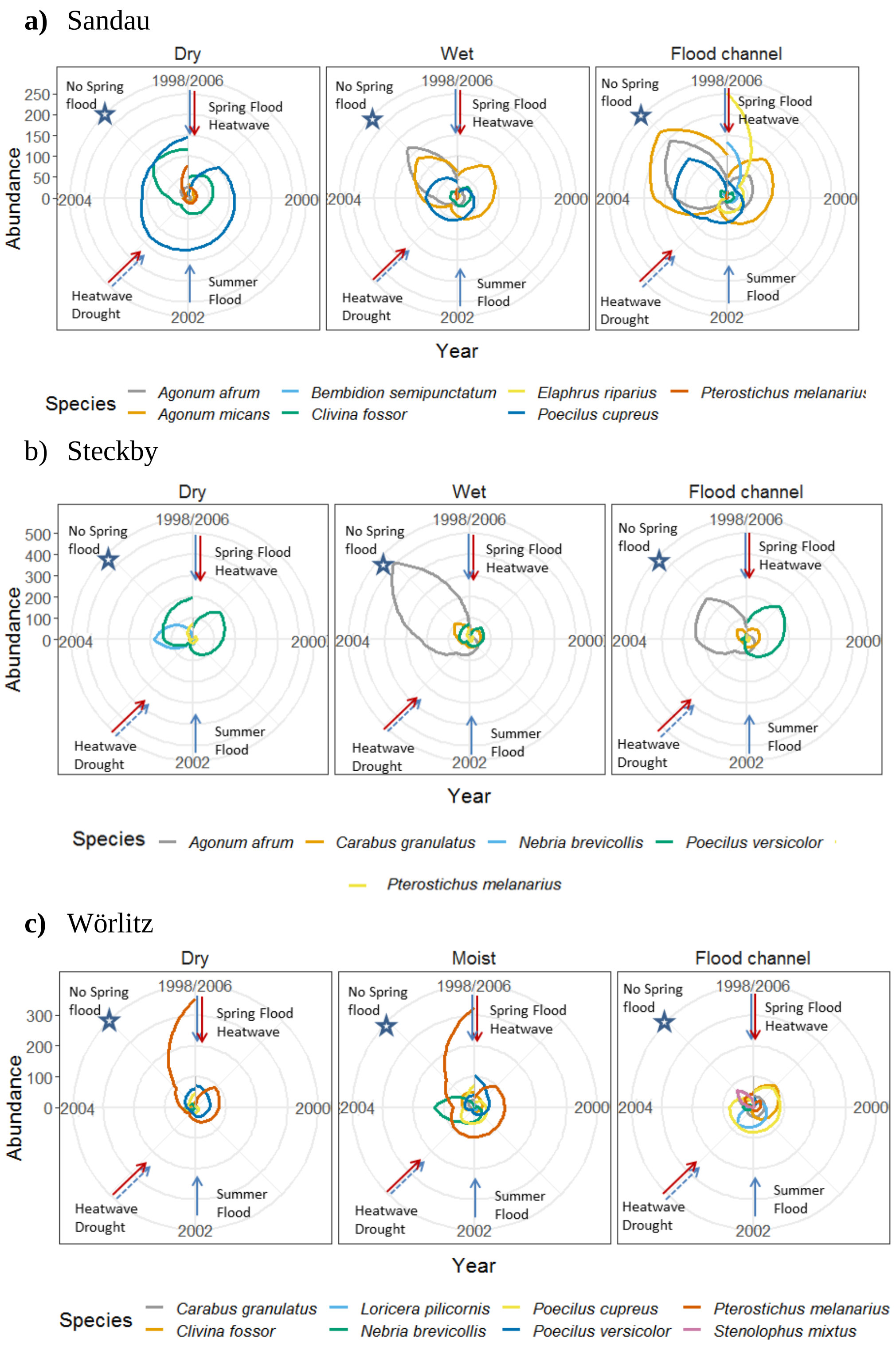

Supplementary Figure S7: Rank clocks showing dominance structure of the carabid beetle community in three plot types at Sandau, Steckby and Wörlitz. Abundance values are shown in the concentric rings.

a) Sandau

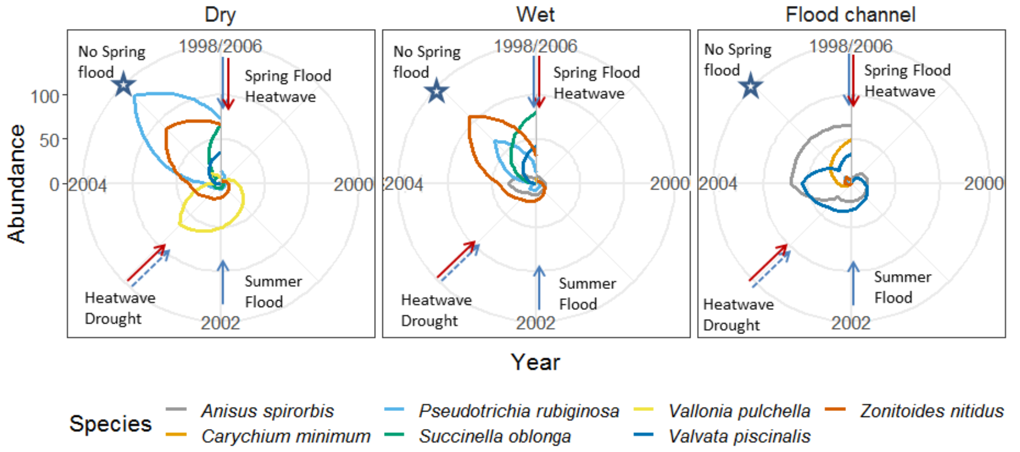

b) Steckby

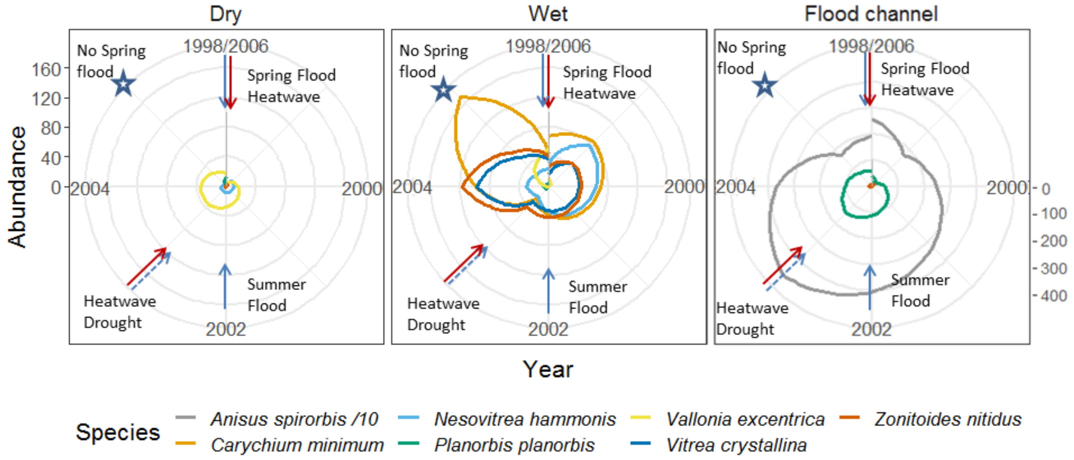

c) Wörlitz

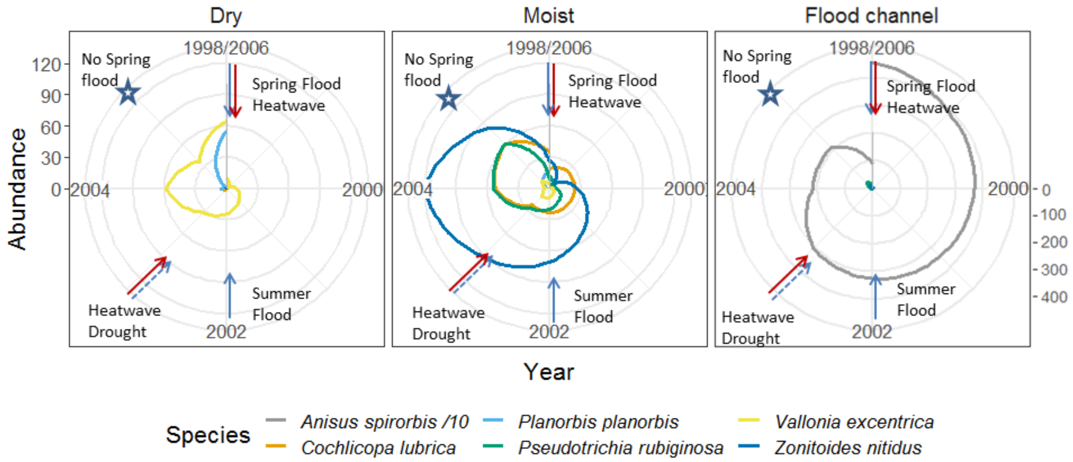

Supplementary Figure S8: Rank clocks showing dominance structure of the mollusc community in three plot types at Sandau, Steckby and Wörlitz. Abundance values are shown in the concentric rings. Note different scales at different sites, and for flood channel plots at Steckby and Wörlitz.

References

Cleveland, R. B., W. S. Cleveland, J. E. McRae, and I. Terpenning. 1990. STL: A Seasonal-Trend Decomposition Procedure Based on Loess. Journal of Official Statistics **6**:3–73.

Foeckler, F., O. Deichner, H. Schmidt, and E. Castella. 2006. Suitability of Molluscs as Bioindicators for Meadow- and Flood-Channels of the Elbe-Floodplains. International Review of Hydrobiology **91**:314-325.
